# Efficiency traps beyond the climate crisis: exploration–exploitation trade-offs and rebound effects

**DOI:** 10.1098/rstb.2022.0405

**Published:** 2023-11-06

**Authors:** Jose Segovia-Martin, Felix Creutzig, James Winters

**Affiliations:** ^1^ School of Collective Intelligence, M6 Polytechnic University (SCI-UM6P), Rabat, 11103 Morocco; ^2^ Complex Systems Institute of Paris Île-de-France (ISCPIF-CNRS), 75013 Paris, France; ^3^ Mercator Research Institute on Global Commons and Climate Change, 10829 Berlin, Germany; ^4^ Technische Universität Berlin, 10623 Berlin, Germany; ^5^ Centre for Culture and Evolution, Department of Psychology, Brunel University London, London, Uxbridge UB8 3PH, UK

**Keywords:** climate change, reinforcement learning, environmental economics, indirect rebound effect, consumption, economic growth

## Abstract

Higher levels of economic activity are often accompanied by higher energy use and consumption of natural resources. As fossil fuels still account for 80% of the global energy mix, energy consumption remains closely linked to greenhouse gas (GHG) emissions and thus to climate change. Under the assumption of sufficiently elastic demand, this reality of global economic development based on permanent growth of economic activity, brings into play the Jevons Paradox, which hypothesises that increases in the efficiency of resource use leads to increases in resource consumption. Previous research on the rebound effects has limitations, including a lack of studies on the connection between reinforcement learning and environmental consequences. This paper develops a mathematical model and computer simulator to study the effects of micro-level exploration–exploitation strategies on efficiency, consumption and sustainability, considering different levels of direct and indirect rebound effects. Our model shows how optimal exploration–exploitation strategies for increasing efficiency can lead to unsustainable development patterns if they are not accompanied by demand reduction measures, which are essential for mitigating climate change. Moreover, our paper speaks to the broader issue of efficiency traps by highlighting how indirect rebound effects not only affect primary energy (PE) consumption and GHG emissions, but also resource consumption in other domains. By linking these issues together, our study sheds light on the complexities and interdependencies involved in achieving sustainable development goals.

This article is part of the theme issue ‘Climate change adaptation needs a science of culture’.

## 1. Introduction

Economic growth is often associated with an increase in energy use [1–4] and resource consumption [5,6]. As fossil fuels still account for 80% of the global energy mix [7,8], energy consumption remains closely linked to greenhouse gas (GHG) emissions and thus to climate change. In this context, the goal of sustainability is to ensure long-term habitation on the Earth by preserving natural resources and maintaining ecological balance. This goal has been widely acknowledged by governments and organizations worldwide. The United Nations' Sustainable Development Goals [9] have set the target to implement energy efficiency improvements in order to promote sustainable development. However, efforts to achieve this goal, through technical innovations motivated by increased efficiency, is often frustrated owing to the rebound effect [10–12]. These rebound effects occur when an increase in resource and energy efficiency is offset by adverse behavioural responses, leading to increased consumption rather than decreased consumption [13].

Under the assumption of sufficiently elastic demand, this reality of global economic development based on permanent growth of economic activity, brings into play the Jevons Paradox, which hypothesizes that increases in the efficiency of resource use leads to increases in resource consumption [14]. Surprisingly, policy makers remain relatively unaware of these issues, with governments, green-focused political parties, and non-governmental organizations tending to believe that efficiency gains lower consumption and thus mitigate deleterious environmental impacts. Yet, when resource and energy efficiency is offset by adverse behavioural responses, the actual savings in energy use, emissions or other environmental impacts are lower than expected [12,15–19].

Recent evidence demonstrates that absolute decoupling is possible. Eighteen developed nations feature reductions of GHG emissions accompanied by growing gross domestic product (GDP) [20]. Many of these reductions are largely driven by an increasing share of renewable energy in the electricity mix. This is in accordance with the predictions of environmental Kuznets curve (EKC), which posits a nonlinear and non-monotonic relationship between economic and environmental factors: economic growth is initially concomitant with environmental degradation until the average income reaches a tipping point and environmental degradation then begins to decrease [21]. However, this trend is not pervasive and not fast enough to make a difference to break increasing global GHG emission trajectories [22]. Other analyses suggest that renewables energies, while underestimated in their potential [23], cannot completely substitute for fossil fuels before 2050 if not accompanied by global reductions in energy demand [24,25]. Furthermore, even with the most positive outlook, other studies question the feasibility of completely breaking the link between GDP and resource consumption [26]. Similarly, a 2020 review of 835 empirical studies reached a similar conclusion: solely relying on decoupling is insufficient to meet climate goals and reduce resource consumption in absolute terms [27]. In this context, carbon emissions are not consistently decreasing according to the EKC, and there is little consensus on whether the EKC with respect to carbon dioxide (CO_2_) emissions has been validated [28,29].

The quantification of rebound effects is commonly accomplished by measuring CO_2_ emissions, GHG emissions or energy use. The magnitude of the estimated rebound effects, measured as GHG emissions, has been demonstrated to vary considerably. Although rebound effects ranging from zero to 100% are the most common, backfire effects (rebound effects greater than 100%) are also frequently observed [13]. Rebound effects can be broken down into direct and indirect rebound effects, particularly at the consumer level [12,15,30]. A direct rebound effect occurs when an increase in the efficiency of energy service provision leads to an increase in the demand for such a service by consumers. For example, when a household replaces their less energy-efficient boiler with a more energy-efficient one, they may enjoy lower heating costs and consequently choose to maintain a higher room temperature, leading to a direct rebound effect. By contrast, an indirect rebound effect occurs when consumers react to an energy efficiency improvement or a sufficiency-related behavioural change by increasing consumption in another area. For instance, they may use the cost savings from a new, more energy-efficient boiler to fund a holiday abroad, which, in turn, generates increased emissions [30].

Prior research has reported a significant variation in the estimated rebound effect (%) [30]. For example, Lenzen & Dey [31] reported a rebound effect of 45–123% in food and heating, Alfredsson [32] reported 7–300% in food, travel and utilities, Brannlund *et al*. [33] 120–175% in transport and utilities, Mizobuchi [34] 12–38% in transport and utilities, Kratena & Wuger [35] 37–86% in transport, heating and electricity, Druckman *et al*. [36] 7–51% in transport, heating and food, Thomas & Azevedo [18,19], 7–25% in transport and electricity, and Murray [37] 4–24% in transport and lighting. This existing body of research reveals a substantial variation in regions, resource domains, metrics and rebound effects. Consequently, there is a pressing need for models that can effectively estimate the values of unknown exploration–exploitation parameters, tailored to fit observed data in empirical studies. However, the current lack of suitable models to estimate parameters and rewards specifically for rebound effects presents a significant gap in the field. To illustrate this, consider a scenario where a society invests in technification while experiencing a rebound effect. In such a context, it becomes imperative to ascertain whether the society is at risk of depleting its energy or material resources. Therefore, the key questions arise: for a given level of investment in technification and rebound effect, how can we determine whether a society is at risk of depleting its energy or material resources? How can we identify the historical periods wherein technological progress should be accompanied by demand restriction policies, as well as those periods where such policies may not be necessary? The scarcity of models looking at these theoretical issues hinders the generation of hypotheses pertaining to interdependencies and feedback mechanisms among resources, and their consequential impact on the global system. Furthermore, the absence of adequate tools impedes the evaluation of cascading effects resulting from changes in one domain on others. To address some of these limitations, here we propose an exploration–exploitation model that offers a parameter estimation tool for different given rebound effect levels. By providing a distinct representation of three sequential resource domains and their corresponding dynamics, our model aims to enable researchers and policy makers to theoretically explore and understand the cascading effects triggered by rebound effects.

Previous studies on the relationship between how people make decisions and their impact on the environment have shortcomings. There is a limited amount of research that looks at the connection between psychological processes and environmental consequences [13]. The theory of moral licensing [38–40] offers a promising starting point and initial evidence of psychological-based rebounds. However, while there is a significant amount of research on licensing effects in relation to climate-related behaviour, studies that specifically measure the extent of spillover effects on energy consumption or emissions are limited. Furthermore, as Reimers *et al*. [13] suggest, research on rebound effects tends to only examine within-domain [41,42] or cross-domain effects (e.g. [43]), but not both. Additionally, there is a lack of modelling research that formalizes and quantifies the negative impact of individual's actions on resource consumption at the systemic level.

Exploration–exploitation models are a class of reinforcement learning models that provide a framework for balancing the exploration of new strategies with the exploitation of currently effective ones [44–47]. In decision making, exploration refers to the process of trying out new options or strategies, while exploitation refers to the process of maintaining the best-known option or strategy currently available. Exploration–exploitation models can provide a useful framework for studying issues related to consumption and sustainability by treating efficient resource utilization as a search problem where populations can either exploit known solutions or explore for more efficient opportunities elsewhere. These models can help balance the trade-off between trying out new methods to conserve resources and using currently effective methods [48]. Moreover, exploration–exploitation models can also be applied to the analysis of consumption behaviour [49], which is a crucial factor in the promotion of sustainable consumption patterns. However, models of reinforcement learning applied to resource consumption and environmental conservation are relatively rare, and their use has been directed more towards social learning and optimization [44,50]. Studies have demonstrated that exploration–exploitation models are valuable in examining resource consumption and sustainability, because they can help to understand how agents, such as individuals or organizations, make decisions about allocating resources in uncertain environments [51,52]. By studying these models, researchers can gain insight into how different factors, such us resource scarcity or regulatory policies, affect resource consumption and sustainability.

In this paper, we ask: what are the global implications of exploration–exploitation dynamics on the long-term trade-off between resource efficiency and consumption across domains? Using a combination of mathematical and computational modelling, where we study the aggregated effects of micro level strategies on efficiency, consumption and sustainability, our model allows us to investigate different levels of direct and indirect rebound effects across resource domains. We formalize the Jevons' Paradox at a systemic level, demonstrating how efficiency-driven decision-making strategies can lead to unsustainable development patterns. Our results speak to questions such as whether the climate and resource consumption crisis can be solved by increased efficiency alone, or under what conditions pursuing efficiency without demand adjustments could become a trap that leads to environmental collapse.

## 2. Methods

We first describe the exploration–exploitation dynamic and explain how we compute efficiency. Next, we outline the three sequential resource domains incorporated in the model. We then describe how we compute resource consumption at the population level as well as the computation of existing resources and the sustainability index. Finally, we present a table with the combinations of parameter values examined.

### (a) The model

The exploration–exploitation dynamic:

We consider a population of agents in which agents must decide at each time step between two possible complementary development strategies:

(i) exploration, which allows agents to improve their current knowledge by searching for new solutions, allowing them to make more informed decisions in the future; or
(ii) exploitation, which refers to the use of already existing solutions. This strategy leads to efficiency stagnation.

Let us consider a vector of possible actions *K* = {1, … ,*k*} where *K* ∈ *N*^+^. We model action selection using a *N*-armed bandit problem that consists of a number of real distributions of efficiency *E* = {*e*_1_, … , *e_k_*}, each of them associated with a reward *M* = *μ*_1_, … , *μ_k_*. We assume that agents have a strong preference for maximizing efficiency so that when they find a more efficient action they receive a higher reward. The initial probability distributions of the efficiency corresponding to each action are different and unknown to the agents. The efficiency of an action chosen by a given agent after *T* time steps is given by the following equation:

$$E_{akt}=1-(T\mu^{*}-\overset{T}{\underset{t=1}{\sum }}\mu_{akt}),$$

where μ* stands for the maximal reward mean and *μ_atk_* is the reward obtained by an agent when using action *k* at time *t*. Since the real efficiency distributions of the system are predefined at the outset, the maximum efficiency of the model is reached when agents obtain the maximum reward. This will allow us to relate the consequences of the maximization model to the consumption of resources in different rebound effect scenarios.

In order for agents to decide which action to choose, actions have a value. The value of an action is defined as the expected efficiency of that action out of the set of all possible actions. Since the agent does not know the value of selecting an action, we use the sample mean to estimate the expected efficiency:

$$E(E_{akt})=\frac{\sum_{t=1}^{T}\mu_{akt}}{n_{kt}},$$

where *n_kt_* is the number of times action *k* was taken before time *t*. This function allows agents who decide to exploit their current knowledge to choose the action that has the most efficiency associated with it, namely max *k_t_*.

We use a simple algorithm to balance exploration and exploitation, in such a way that the mathematical optimization can yield locally optimal solutions that approximate a globally optimal solution. At each time step, agents take either the action that seems to be optimal (max *k_t_*) with probability (1 − ε) (i.e. exploitation), or a random action with probability ε (i.e. exploration). In this context, ε = 0 means full exploitation, and ε = 1 means full exploration. That is to say, the optimization choice function *f(x)* is given by:

$$f(x)=\{\begin{array}{ccc} \max\;k_{t}, & \mathrm{with} & \;p=1-\varepsilon \\ k_{t}∼U(\left[1,k\right]), & \mathrm{with} & \;p=\varepsilon \end{array},$$

where max *k_t_* is the optimal action according to the observed data at time *t*, and *k_t_* ∼ *U*([1, *k*]) is a uniform random choice that takes values in *K* = [1, … ,*k*].

### (b) Resource domains

The proposed mathematical model comprises three sequential resource domains, each representing a distinct resource and its corresponding dynamics. *Resource domain 1* pertains to primary energy (PE) and is quantified in terms of the existing resource stock denoted as gross available energy, measured in abstract units (a.u.) that can be mapped into physical units (e.g. in kilowatt-hour, kWh), and the mean consumption level denoted as the overall PE consumption. *Resource domain 2* represents target good 2, a natural resource that is initially consumed owing to the indirect rebound effect of the first domain, and is quantified in abstract numerical units. Finally, *resource domain 3* captures the consumption dynamics of target good 3, which starts to be used following an improvement in consumption efficiency of target good 2, and is also quantified in abstract numerical units. These resource domains enable the modelling of the interdependencies and feedback mechanisms between the resources and their impact on the overall system, irrespective of the specific physical units used. The distinct representation of each resource domain facilitates the assessment of the cascading effects of changes in one domain on the other domains, which is a crucial feature for policy formulation and scenario analysis.

### (c) Resource consumption

At each time step, the resources consumed from the primary resource domain *C*_1_ by a typical agent with efficiency *E_a_* are computed as the baseline *per capita* consumption plus the difference between the aggregate consumption owing to the direct rebound effect and the unrealized consumption owing to efficiency gains:

$$C_{1}=\beta_{1}+E_{a}D_{1}-(1-D_{1})E_{a},$$

where *β*_1_ stands for the baseline *per capita* consumption in resource units, *E_a_* stands for agent's real efficiency, and *D*_1_ is the marginal direct rebound level measured as additional number of resource units consumed for each unit of efficiency gain. This means that when the rebound *D*_1_ is less than 0.5, efficiency gains compensate for the rebound effect (i.e. sustainable scenario). When *D*_1_ is greater than 0.5, efficiency gains cannot compensate for the rebound effect (i.e. Jevons Paradox scenario). When *D*_1_ is 0.5, we have a neutral model, where the actual resource savings are equal to the increase in usage (according to some classical measures this corresponds to scenarios where the rebound effect (*r*) is at 100%).

Indirect rebound effects at the consumer level occur when potential savings (e.g. lower GHG emissions owing to less consumption of fossil resources) resulting from the use of more efficient technologies or more responsible consumption in one consumption domain are partially or fully offset by consumption in other domains. We model indirect rebound effects as the additional resource consumption in a subsequent domain owing to a shift of resource consumption away from the primary domain as a consequence of efficiency gains (e.g. efficiency gains associated with a fall in the relative price of secondary domain resource consumption). We assume that for each discrete jump by one unit in efficiency in *E* = {*e*_1_, … ,*e_k_*}, agents are able to start consuming resources from a subsequent domain *n* + 1. The consumption function is governed by the following expression:

$$f(C)=\{\begin{array}{ccc} & \mathrm{if} & E_{a}<e_{1}\{\begin{array}{c} C_{1}=\beta_{1}+E_{a}\;D_{1}\;-(1-D_{1}\;)E_{a}\;\\ C_{2}=\beta_{2}\\ \ldots \\ C_{n}=\beta_{n} \end{array}\\ \mathrm{else} & \mathrm{if} & e_{1}<E_{a}\leq e_{2}\{\begin{array}{c} C_{1}=\beta_{1}+E_{a}\;D_{1}\;(1-I_{1})-(1-D_{1}\;)E_{a}\;\\ C_{2}=\beta_{2}+E_{a}\;D_{2}\;I_{1}-(1-D_{2}\;)E_{a}\;\\ \ldots \\ C_{n}=\beta_{n} \end{array}\\ & & \ldots \\ \mathrm{else} & \mathrm{if} & \;e_{k-1}\;<E_{a}\leq e_{k}\{\begin{array}{c} C_{1}=\beta_{1}+E_{a}\;D_{1}\;(1-I_{1})-(1-D_{1}\;)E_{a}\;\\ C_{2}=\beta_{2}+E_{a}\;D_{2}\;I_{1}\;(1-I_{2})-(1-D_{2}\;)E_{a}\;\\ \ldots \\ C_{n}=\beta_{n}E_{a}\;D_{n}\;I_{1}I_{2}\ldots I_{n-1} \end{array}, \end{array}$$

where *I_n_* represents the share of resource consumption owing to rebound effects that are consumed in the subsequent domain *n* + 1 as a consequence of the indirect rebound effect. For each resource domain, an indicative sustainability index *i* can be computed at each time step by *i* = *β_n_*/*C_n_*, reflecting baseline needs at *t*_0_ met for each unit of resources consumed. When *i* = 1, it means that for each unit of resources consumed, we maintain the needs of one individual. When *i* < 1, it means that more resources are consumed than the population would need to meet its basic needs. When *i* > 1, it means that fewer resources are consumed than the population would need to cover their basic needs.

### (d) Existing resources

We compute existing resources *X* in each resource domain *n* at each time step *t* as the existing resources minus the resources consumed times the replenishment rate of the remaining resources:

$$X_{nt}=\;(X_{n(t-1)}-\;C_{nt})+(X_{n(t-1)}-\;C_{nt})\alpha_{n},$$

which can be grouped as:

$$X_{nt}=\;(X_{n(t-1)}-\;C_{nt})(1+\alpha_{n}\;),$$

where *α_n_* stands for resource units replenished per unit of existing resources at each time step.

We run 1000 simulations for each of the parameter combinations shown in table 1.

**Table 1.** Parameters and state variables.

parameter	symbol	number of levels	value(s)
time, time steps	*T*, *t*	10 000	0 to 10 000 in steps of 1
population size	*A*	2	100, 1000
typical agent	*a*		
actions	*K* = {1, … , *k*}	4	
strategies	exploration and exploitation	2	
probability of choosing to explore	*ε*	5	0, 0.01, 0.05, 0.1, 0.5
efficiency distributions	*E* = {*e*_1_, … , *e_k_*}	4	0 to 3 in steps of 1
real efficiency of agent *a* with action *k* at time *t*	*E_akt_*		
expected efficiency of agent *a* with action *k* at time *t*	*E*(*E_akt_*)		
rewards	*M* = *μ*_1_, … , *μ_k_*	4	0 to 3 in steps of 1
baseline consumption	*β*	1	0.1
total consumption	*C*		
existing resources	*X*		10 000
replenishment rate	*α*	2	0.01, 0.001
direct rebound level	*D*	11	0 to 1 in steps of 1
rebound effect	r	11	0 to 2 in steps of 2
indirect rebound level	*I*	11	0 to 1 in steps of 1
correction factor	*c*	1	10
resource domain	*n*	3	

## 3. Results

We first consider outputs from the standard model without indirect rebound effects. We then simulate scenarios with indirect rebound effects across three consecutive resource domains. Finally, we analyse scenarios with partial indirect rebound effects subject to low replenishment rate conditions.

### (a) Direct rebound effect

**Figure 1.**
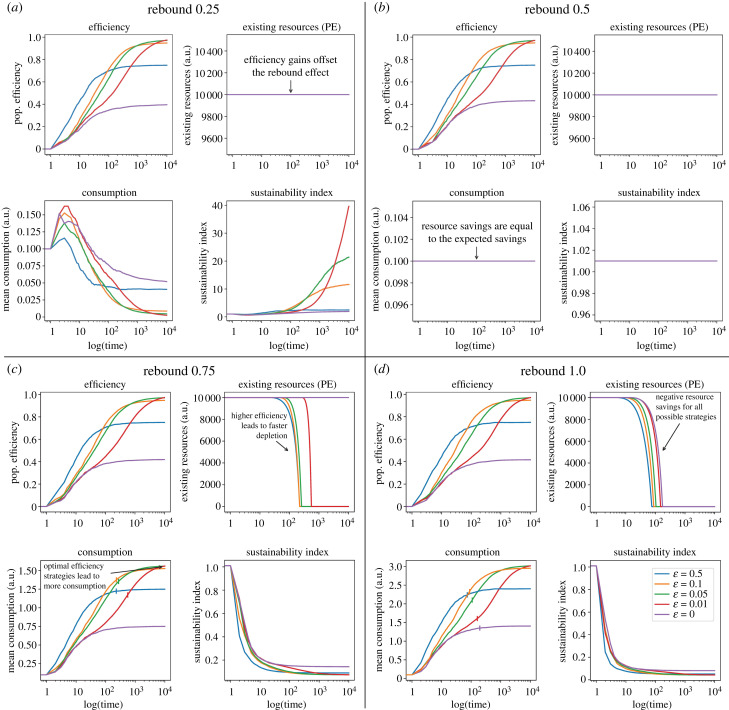
Direct rebound effects. Trajectories for efficiency, existing stock of primary energy (PE) resources denoted as gross available energy, and average PE consumption for different levels of direct rebound effect and probability of exploration (*ε*). We use abstract numerical units that can be mapped into physical units (e.g. gross available energy and mean PE consumption expressed in kilowatt-hour, kWh). Simulations using the following parameter values: *N* = 100; *t* = 10 000; *ε* = [0, 0.01, 0.05, 0.1, 0.5]; *β*_1_ = 0.1; *X*_1(*t*=0)_ = 10 000; *α*_1_ = 0.01; *D*_1_ = [0.25, 0.5, 0.75, 1]; *I*_1_ = 0; *M* = [0, 1, 2, 3]. The vertical marks intersecting the mean consumption lines represent the point of resource exhaustion. The subsequent projections beyond this point illustrate the consumption trends in the absence of resource constraints. (*a*) Rebound level 0.25: this corresponds to a situation where the actual resource savings are higher than the expected savings. (*b*) Rebound level 0.5: this corresponds to a situation where the actual resource savings are equal to the expected savings owing to efficiency gains. (*c*) Rebound level 0.75: the actual resource savings are less than expected savings. (*d*) Rebound level 1.0: in this scenario, there is no real saving of resources because all the efficiency gain is transformed into rebound effect.

We first consider the effects of exploration–exploitation strategies on efficiency, consumption and sustainability, assuming only direct rebound effects. Here we show simulation results for the parameter values indicated in figure 1.

When the marginal direct rebound level *D* is less than 0.5 (figure 1*a*), there is no resource depletion. In our model, this corresponds to a situation where the actual resource savings are higher than the expected savings. Efficiency gains offset the rebound effect. In this scenario, as efficiency increases, average consumption in the primary resource domain goes down and sustainability index grows over time. The sustainability index grows faster in the scenarios with optimal efficiency strategies (i.e. 0.01 ≤ *ε* ≤ 0.1, strategies with relatively high exploration levels, but not too high to prevent exploitation). This scenario is equivalent to a scenario where the consumption of fossil resources decreases over time, leading to a reduction in relative emissions, but not necessarily to an absolute reduction in the concentration of GHGs in the atmosphere.

When *D* = 0.5 (figure 1*b*), this corresponds to a situation where the actual resource savings are equal to the expected savings owing to efficiency gains. In this scenario, there is no resource depletion, there is constant average consumption and the sustainability index remains constant at 1. This model corresponds to a neutral model. This corresponds to a 100% direct rebound effect (*r*) according to the classical measurement, i.e. *r* = 1 − (*E* − *D*) = 1.

When 0.5 < D ≤ 1 (e.g. figure 1*c*), the actual resource savings are less than expected savings. In this scenario, optimal efficiency strategies increase the probability of resource depletion. Paradoxically, there is no resource depletion in scenarios with lower efficiency (*ε* = 0), because the rebound effect does not overtake the resource replenishment rate. The higher the efficiency, the faster resources are depleted. Consumption increases over time in most scenarios. The sustainability index decreases over time. In general, in this scenario, backfire depends on the amount of available resources, the rate of resource replenishment and population size.

When *D* = 1 (figure 1*d*), in this scenario there is no real saving of resources because all the efficiency gain is transformed into rebound effect. This situation captures the Jevons Paradox for all possible exploration–exploitation strategies. The higher the efficiency, the faster the probability of resource depletion. Consumption goes up over time and the sustainability index decreases over time.

### (b) Indirect rebound effect

To expand our model, we now consider the effects of exploration–exploitation strategies when efficiency gains (e.g. more efficient technologies) are partially offset by behavioural responses in other resource domains. This aims to capture scenarios where potential savings in one domain (e.g. lower consumption of fossil fuels) lead to a corresponding increase in the consumption of resources in other domains. We assume that for each discrete jump by one unit in efficiency, agents can start consuming resources from a subsequent domain.

**Figure 2.**
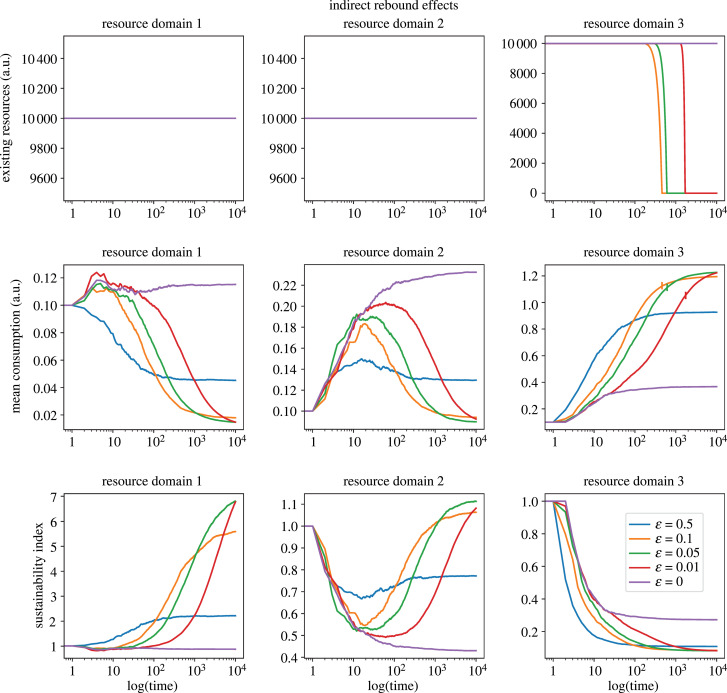
Indirect rebound effects. Trajectories for efficiency, existing resources and consumption across three sequential resource domains. Resource domain 1 pertains to primary energy (PE) and is quantified in terms of the existing resource stock denoted as gross available energy, measured in abstract units that can be mapped into physical units (e.g. in kilowatt-hour, kWh), and the mean consumption level denoted as the PE consumption. Resource domain 2 represents target good 2, a natural resource that is initially consumed owing to the indirect rebound effect of the first domain, and is quantified in abstract numerical units. Resource domain 3 captures the consumption dynamics of target good 3, which starts to be used following an improvement in consumption efficiency of target good 2, and is also quantified in abstract numerical units. Simulations using the following parameter values: *N* = 100; *t* = 10 000; *ε* = [0, 0.01, 0.05, 0.1, 0.*5*]; *β*_1_ = *β*_2_ = *β*_3_ = 0.1; *X*_1(*t*=0)_ = *X*_2(*t*=0)_ = *X*_3(*t*=0)_ = 10 000; *α*_1_ = *α*_2_ = *α*_3_ = 0.01; *D*_1_ = *D*_2_ = *D*_3_ = 0.75; *I*_1_ = *I*_2_ = *I*_3_ = 0.75; *M* = [0, 1, 2, 3]. The vertical marks intersecting the mean consumption lines represent the point of resource exhaustion. The subsequent projections beyond this point illustrate the consumption trends in the absence of resource constraints. Optimal efficiency strategies ultimately lead to resource exhaustion in domain 3, where the manifestation of transferred backfire effects becomes evident.

Indirect rebound effects that lead to backfire, capturing the Jevons Paradox at the systemic level, can be observed in simulations with *D* > 0.5 (*r* > 100%). To illustrate this phenomenon, we present simulations with *D* = 0.75 and *I* = 0.75 in figure 2. The simulations involve three distinct resource domains, each representing different types of resources, which are sequentially consumed as efficiency increases with each efficiency leap. Initially, agents consume from resource domain 1, but as their efficiency improves, they shift consumption to resource domain 2. Similarly, upon reaching the next efficiency threshold, they transition to resource domain 3. Consequently, for strategies with *ε* > 0, average consumption in each resource domain increases until reaching tipping points, beyond which consumption starts to decline. The timing of these tipping points depends on the level of efficiency attained, with higher efficiency levels reaching them earlier. This is attributed to the fact that as efficiency improves, agents divert their consumption to subsequent resource domains, thereby mitigating exploitation pressure in the primary domains.

It is noteworthy that efficiency gains slow down over time, resulting in diminishing returns for all exploration–exploitation strategies, as illustrated in figure 1. For instance, strategies with *ε* = 0.01 show a rapid increase in actual (normalized) efficiency from 0 to approximately 0.2 within 10 time steps. However, it takes an additional 100 time steps to achieve an efficiency of 0.4. This explains why populations reach consumption tipping points quickly in the first domain, but takes longer to reach a tipping point in the second and third domains (figure 2, mean consumption). As the system approaches its theoretical limits, further optimizations become more challenging, and as efficiency improvements become progressively more challenging, populations spend more time-consuming resources in each subsequent domain until the next efficiency threshold is reached. Consequently, the accumulation of total consumption over time intensifies, increasing the likelihood of resource stock depletion.

Indirect rebound effects, by shifting consumption from the primary domain to subsequent domains, alleviate exploitation pressure in the primary domains. Figure 2 illustrates this phenomenon in domain 1, and particularly in domain 2, where mean consumption initially rises and then declines, while the sustainability index decreases and then increases. However, under the assumption of equivalent resource replenishment levels, this merely displaces the unsustainable exploitation pattern to subsequent domains. Simulation results reveal that optimal efficiency strategies ultimately lead to resource exhaustion in domain 3, where the manifestation of transferred backfire effects becomes evident. This resource exhaustion is visually represented by vertical marks intersecting with the mean consumption lines, indicating that expected consumption trends cannot be sustained in these scenarios (figure 2, mean consumption, resource domain 3). Notably, strategies that reach optimal efficiency levels faster (i.e. 0.05 ≤ ε ≤ 0.1) exhaust resources shortly after *t* = 400, while strategies with slower progress towards optimal efficiency levels (i.e. *ε* = 0.01) deplete resources later, around *t* = 2000. On the other hand, strategies that fail to attain optimal efficiency (i.e. *ε* = 0 and *ε* = 0.5) do not reach consumption levels significant enough to deplete the available resources. This observation exemplifies the essence of the Jevons Paradox at a systemic level and serves as a showcase, highlighting that while optimal exploration–exploitation strategies can enhance efficiency, their implementation without accompanying demand reduction measures can result in unsustainable development patterns.

### (c) Partial rebounds

**Figure 3.**
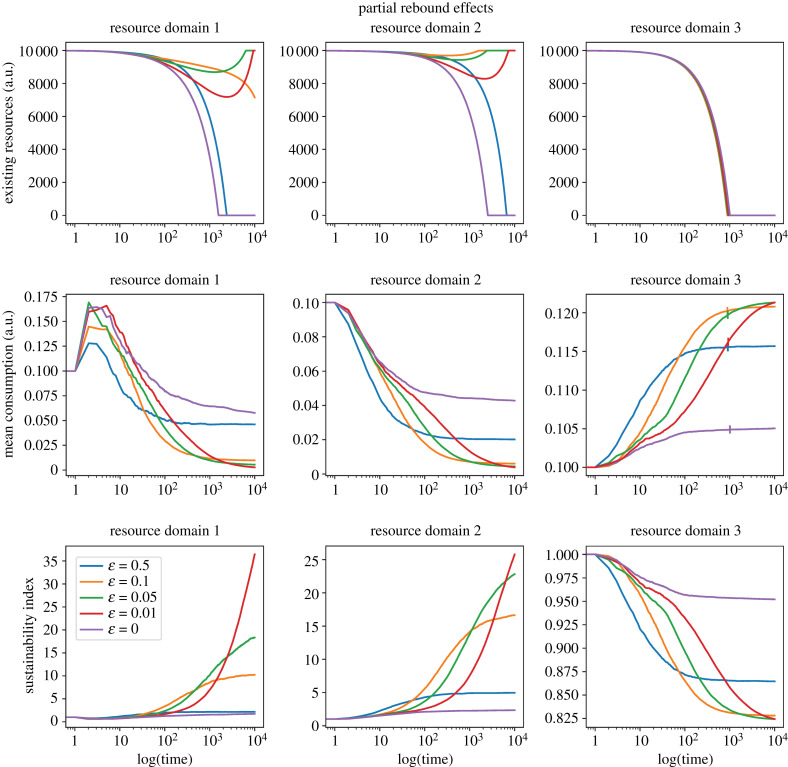
Partial rebound effects. Trajectories for efficiency, existing resources and consumption across three sequential resource domains. Simulations using the following parameter values: *N* = 100; *t* = 10 000; *ε* = [0, 0.01, 0.05, 0.1, 0.5]; *β*_1_ = *β*_2_ = *β*_3_ = 0.1; *X*_1(*t*=0)_ = *X*_2(*t*=0)_ = *X*_3(*t*=0)_ = 10 000; *α*_1_ = *α*_2_ = *α*_3_ = 0.0001; *D*_1_ = *D*_2_ = *D*_3_ = 0.25; *I*_1_ = *I*_2_ = *I*_3_ = 0.25; *M* = [0, 1, 2, 3]. The vertical marks intersecting the mean consumption lines represent the point of resource exhaustion. The subsequent projections beyond this point illustrate the consumption trends in the absence of resource constraints. Partial rebounds can contribute to resource depletion when replenishment levels are insufficient.

Partial rebounds (0 < *D* < 0.5, i.e. 0 < *r* < 100%) can also contribute to resource depletion when replenishment levels are insufficient. We present simulations for partial rebound scenarios where the actual resource savings are lower than expected owing to a 50% direct rebound effect (*D* = 0.25) and 50% indirect rebound effects across domains (*I* = 0.25). We analyse scenarios where resources are scarcely renewable, with a replenishment rate set at 0.0001 (figure 3).

In this context of partial rebound effects, exploration–exploitation strategies that best maximize efficiency can compensate for the increase in consumption resulting from the rebound effect in the primary and secondary domains. Despite the initial decline in the level of existing resources owing to low initial efficiency and a low replenishment rate, these strategies eventually enable the ecosystem to restore the original resource levels. However, it is important to note that the indirect rebound effect propagates consumption to subsequent domains, leading to an inevitable collapse of the system in the final resource extraction domain when replenishment rates are exceedingly low.

## 4. Discussion

In this study, we investigate the effects of exploration–exploitation strategies on efficiency, consumption and sustainability, taking into account different levels of direct and indirect rebound effects. Our model successfully formalizes the Jevons' Paradox at a systemic level, demonstrating how efficiency-driven decision-making strategies can lead to unsustainable development patterns. Our results provide a comprehensive understanding of the behavioural mechanisms driving rebound effects and the quantification of the consequences of these mechanisms in terms of resource consumption and sustainability. Furthermore, it highlights the importance of considering both efficiency improvements and demand-side solutions for achieving sustainable development goals. While improving efficiency is crucial in reducing relative resource consumption and emissions, it may not be enough on its own to achieve absolute reduction. As proposed in a large number of previous studies, it is also essential to address demand-side factors by reducing aggregate resource use, energy demand and emissions, and implementing redistribution policies, cap-and-trade schemes or carbon taxes [24,53–56]. This approach ensures that sustainable development goals can be met in a comprehensive and holistic manner.

The relationship between long-term optimal strategies in terms of efficiency and sustainability is complex, as optimal efficiency strategies do not always align with reductions in total PE consumption, GHG emissions or material consumption. Specifically, we found that when *D* exceeds 0.5, efficiency gains cannot compensate for the rebound effect, leading to increased consumption and increased probability of resource depletion. Conversely, when *D* is below 0.5, efficiency gains do compensate for the rebound effect, leading to decreased consumption. As such, policy recommendations should prioritize demand restraint measures in scenarios where the quantified rebound effect is high (*D* > 0.5 or *r* > 100%), and particularly in cases where knowledge on the rebound effect is not sufficiently reliable. This holds particularly true in situations where the exploration–exploitation balance approaches optimality. For example, it may be advisable to consider demand reduction policies and practices in areas where there are high rebound effects, low replenishment rates and high investment in efficient technologies. As behavioural responses to such measures and rebound effects may vary depending on resource domains and income levels [12,13,15–19], we emphasize the importance of tailoring measures to specific contexts and income levels. Overall, our results stress the need for careful consideration of the rebound effect and behavioural responses in policy decisions related to resource efficiency and sustainability.

The United Nations' Sustainable Development Goals [9] include the aim to implement energy efficiency improvements to promote sustainable development. However, as previous studies have shown, the effort to achieve these goals through technical innovations [10–12] or consumer behaviours [36,57] is often thwarted by rebound effects. Our study highlights the potential role of decision-making strategies in driving rebound effects. Consumer studies, such as those by Catlin & Wang [41] and Noblet & McCoy [58], have shown that consumers may feel a moral license in one product or consumption domain, leading to less environmentally conscious behaviour in the same or another domain. Consistent with this finding, our model shows that individuals may be motivated by virtuousness to pursue exploration strategies, increasing resource consumption. However, previous research has limitations, such as a scarcity of studies that examine the relationship between psychological processes and environmental consequences [13], and a lack of research that formalizes, models and quantifies the negative impact of behavioural responses on resource consumption. Furthermore, studies on rebound effects tend to focus on either within-domain [41,42] or cross-domain effects (e.g. [43]), but not both (e.g. [59,60]). Our study aims to fill these gaps by providing a comprehensive understanding of the exploration–exploitation mechanisms that drive direct and indirect rebound effects within and across domains, and by quantifying the consequences of these mechanisms in terms of resource consumption and sustainability.

One advantage of our model is that it uses abstract numerical units, which allows for easier manipulation and experimentation with the model's parameters. Changing the numerical values of the agents' behaviours and interactions can be done quickly and easily, without having to consider the physical implications of those changes. This can save time and resources in model development and testing. This can make agent-based models more generalizable across different contexts, as the model can be easily adapted to represent different systems by changing the numerical values of the parameters. In this way, the present study introduces a tool capable of theoretically estimating unknown parameters for a broad range of case studies [31] pertaining to diverse areas—i.e. electricity, lighting, transport, heating, food, among others—and resource domains—i.e. GHG-based energy consumption, PE consumption and material consumption metals (non-metallic minerals, biomass and fossil energy carriers). Nevertheless, one of the limitations of models that use abstract numerical units is that it can be challenging to translate the model's output into real-world implications. Representing the complexities of real-world energy and resource systems accurately can be problematic when using abstract numerical units, resulting in unrealistic assumptions or predictions. Moreover, because abstract numerical units do not directly correspond to physical units, it can be challenging to integrate physical laws and constraints into the model, limiting its accuracy and applicability. Therefore, we suggest using caution when drawing conclusions from our model, although we believe it can be valuable in generating hypotheses and informing environmental policy complemented by empirical studies.

The current version of the model assumes movement to new resource domains is unidirectional. This models a scenario where a population initially consumes one resource (e.g. wood) and then moves to a newly discovered resource (e.g. coal). However, it seems plausible that there are situations where a society consumes a new resource but moves back to the previous resource. We should expect such situations to emerge when resources vary on some perceivable dimensions, such as differences in the rate of replenishment or the ease with which resources are extracted, and societies use this information to make decisions about what resources they exploit at any given time. If societies, and the individuals within them, are active decision-makers who have some autonomy over what resources they exploit, then one solution is for populations to employ dynamic equilibrium strategies; here, movement between resources is monitored and updated depending on the rate of replenishment and current consumption levels. Future research can contrast such extensions with our existing model to see if they depart and result in fundamentally different long-term dynamics.

To conclude, our study also provides an important avenue for future research to further explore the propagation of consumption to downstream domains under partial rebound effects. In the literature, the study of partial rebound effects under low replenishment rate conditions is not well established. However, it is important to consider that if the extraction rate of a resource exceeds the replacement rate, the resource will become finite and eventually depleted (e.g. [61]). While initial resource levels may decrease owing to low efficiency and replenishment rates, optimal exploration–exploitation strategies for increasing efficiency have the potential to restore the primary resource domain to its original resource levels. However, it should be noted that indirect rebound effects can propagate the consumption level to downstream domains. For instance, in cases where replenishment rates are extremely low, collapse of the system in the final resource extraction domain may be inevitable if resource demand is not limited, constrained or restrained.

## Data Availability

The code and supplementary materials is available from the Github Repository: https://github.com/School-of-Collective-Intelligence/Jevons-Paradox-and-Cultural-Evolution [62]. A simulation tool can be accessed from the following links: https://jevons-collectiveintelligence.pythonanywhere.com/ or https://jsegoviamartin.pythonanywhere.com/ [63]. This material is also available from the Dryad Digital Repository: https://doi.org/10.5061/dryad.qjq2bvqnk [64] as part of the Climate Change Adaptation Needs a Science of Culture data portal on the Dryad Digital Repository: https://doi.org./10.5061/dryad.bnzs7h4h4 [65].

## References

[1] Stern DI. 2004 Economic growth and energy. Encycl. Energy **2**, 35-51. (10.1016/B0-12-176480-X/00147-9)

[2] Ockwell DG. 2008 Energy and economic growth: grounding our understanding in physical reality. Energy Policy **36**, 4600-4604. (10.1016/j.enpol.2008.09.005)

[3] Kais S, Sami H. 2016 An econometric study of the impact of economic growth and energy use on carbon emissions: panel data evidence from fifty eight countries. Renew. Sust. Energy Rev. **59**, 1101-1110. (10.1016/j.rser.2016.01.054)

[4] Sadraoui T, Hamlaoui H, Youness Z, Sadok BI. 2019 A dynamic panel data analysis for relationship between energy consumption, financial development and economic growth. Int. J. Econ. Fin. Manag. **7**, 20-26. (10.12691/ijefm-7-1-3)

[5] Bringezu S, Schütz H, Steger S, Baudisch J. 2004 International comparison of resource use and its relation to economic growth: the development of total material requirement, direct material inputs and hidden flows and the structure of TMR. Ecol. Econ. **51**, 97-124. (10.1016/j.ecolecon.2004.04.010)

[6] United Nations. 2011 Environment Programme. International Resource Panel, United Nations Environment Programme. Sustainable Consumption, & Production Branch. Decoupling natural resource use and environmental impacts from economic growth. New York, NY: UNEP/Earthprint.

[7] Abas N, Kalair A, Khan N. 2015 Review of fossil fuels and future energy technologies. Futures **69**, 31-49. (10.1016/j.futures.2015.03.003)

[8] Energy Study Institute (EESI), E. A. 2021 Fossil Fuels | EESI. Fossil Fuels | EESI. Retrieved 25 January 2023. See https://www.eesi.org/topics/fossil-fuels/description.

[9] United Nations. 2015 General assembly transforming our world: the 2030 agenda for sustainable development A/RES/70/1. New York, NY: United Nations.

[10] Binswanger M. 2001 Technological progress and sustainable development: what about the rebound effect? Ecol. Econ. **36**, 119-132. (10.1016/S0921-8009(00)00214-7)

[11] Azevedo IM. 2014 Consumer end-use energy efficiency and rebound effects. Annu. Rev. Environ. Resour. **39**, 393-418. (10.1146/annurev-environ-021913-153558)

[12] Santarius T, Soland M. 2018 How technological efficiency improvements change consumer preferences: towards a psychological theory of rebound effects. Ecol. Econ. **146**, 414-424. (10.1016/j.ecolecon.2017.12.009)

[13] Reimers H, Jacksohn A, Appenfeller D, Lasarov W, Hüttel A, Rehdanz K, Balderjahn I, Hoffmann S. 2021 Indirect rebound effects on the consumer level: a state-of-the-art literature review. Cleaner Respons. Consump. **3**, 100032. (10.1016/j.clrc.2021.100032)

[14] Jevons WS. 1866 ‘VII’. The coal question, 2nd edn. London, UK: Macmillan and Company. OCLC 464772008. Retrieved 25 January 2023. See https://oll.libertyfund.org/title/jevons-the-coal-question.

[15] Sorrell S. 2007 The rebound effect: an assessment of the evidence for economy-wide energy savings from improved energy efficiency.

[16] Guerra AI, Sancho F. 2010 Rethinking economy-wide rebound measures: an unbiased proposal. Energy Policy **38**, 6684-6694. (10.1016/j.enpol.2010.06.038)

[17] Chitnis M, Sorrell S, Druckman A, Firth SK, Jackson T. 2013 Turning lights into flights: estimating direct and indirect rebound effects for UK households. Energy Policy **55**, 234-250. (10.1016/j.enpol.2012.12.008)

[18] Thomas BA, Azevedo IL. 2013 Estimating direct and indirect rebound effects for US households with input–output analysis. Part 1: theoretical framework. Ecol. Econ. **86**, 199-210. (10.1016/j.ecolecon.2012.12.003)

[19] Thomas BA, Azevedo IL. 2013 Estimating direct and indirect rebound effects for US households with input–output analysis. Part 2: simulation. Ecol. Econ. **86**, 188-198. (10.1016/j.ecolecon.2012.12.002)

[20] Le Quéré C et al. 2019 Drivers of declining CO_2_ emissions in 18 developed economies. Nat. Clim. Change **9**, 213-217. (10.1038/s41558-019-0419-7)

[21] Yasin I, Ahmad N, Chaudhary MA. 2021 The impact of financial development, political institutions, and urbanization on environmental degradation: evidence from 59 less-developed economies. Environ. Dev. Sustain. **23**, 6698-6721. (10.1007/s10668-020-00885-w)

[22] Lamb WF et al. 2021 A review of trends and drivers of greenhouse gas emissions by sector from 1990 to 2018. Environ. Res. Lett. **16**, 073005. (10.1088/1748-9326/abee4e)

[23] Creutzig F, Agoston P, Goldschmidt JC, Luderer G, Nemet G, Pietzcker RC. 2017 The underestimated potential of solar energy to mitigate climate change. Nat. Energy **2**, 1-9. (10.1038/nenergy.2017.140)

[24] Holechek JL, Geli HM, Sawalhah MN, Valdez R. 2022 A global assessment: can renewable energy replace fossil fuels by 2050? Sustainability **14**, 4792. (10.3390/su14084792)

[25] Creutzig F, Hilaire J, Nemet G, Müller-Hansen F, Minx JC. 2022 Climate change mitigation easier than suggested by models. Authorea Preprints. See https://www.authorea.com/doi/full/10.1002/essoar.10506825.1.

[26] Hickel J, Kallis G. 2020 Is green growth possible? New Polit. Econ. **25**, 469-486. (10.1080/13563467.2019.1598964)

[27] Haberl H et al. 2020 A systematic review of the evidence on decoupling of GDP, resource use and GHG emissions, part II: synthesizing the insights. Environ. Res. Lett. **15**, 065003. (10.1088/1748-9326/ab842a)

[28] Uchiyama K. 2016 Environmental kuznets curve hypothesis, pp. 11-29. Tokyo, Japan: Springer.

[29] Galeotti M, Lanza A, Pauli F. 2006 Reassessing the environmental Kuznets curve for CO_2_ emissions: a robustness exercise. Ecol. Econ. **57**, 152-163. (10.1016/j.ecolecon.2005.03.031)

[30] Chitnis M, Sorrell S, Druckman A, Firth SK, Jackson T. 2014 Who rebounds most? Estimating direct and indirect rebound effects for different UK socioeconomic groups. Ecol. Econ. **106**, 12-32. (10.1016/j.ecolecon.2014.07.003)

[31] Lenzen M, Dey CJ. 2002 Economic, energy and greenhouse emissions impacts of some consumer choice, technology and government outlay options. Energy Econ. **24**, 377-403. (10.1016/S0140-9883(02)00007-5)

[32] Alfredsson EC. 2004 ‘Green’ consumption—no solution for climate change. Energy **29**, 513-524. (10.1016/j.energy.2003.10.013)

[33] Brännlund R, Ghalwash T, Nordström J. 2007 Increased energy efficiency and the rebound effect: effects on consumption and emissions. Energy Econ. **29**, 1-17. (10.1016/j.eneco.2005.09.003)

[34] Mizobuchi K. 2008 An empirical study on the rebound effect considering capital costs. Energy Econ. **30**, 2486-2516. (10.1016/j.eneco.2008.01.001)

[35] Kratena K, Wüger M. 2010 The Full Impact of Energy Efficiency on Households' Energy Demand (No. 356). WIFO Working Papers.

[36] Druckman A, Chitnis M, Sorrell S, Jackson T. 2011 Missing carbon reductions? Exploring rebound and backfire effects in UK households. Energy Policy **39**, 3572-3581. (10.1016/j.enpol.2011.03.058)

[37] Murray CK. 2013 What if consumers decided to all ‘go green’? Environmental rebound effects from consumption decisions. Energy Policy **54**, 240-256. (10.1016/j.enpol.2012.11.025)

[38] Blanken I, Van De Ven N, Zeelenberg M. 2015 A meta-analytic review of moral licensing. Pers. Soc. Psychol. Bull. **41**, 540-558. (10.1177/0146167215572134) 25716992

[39] Miller DT, Effron DA. 2010 Psychological license: when it is needed and how it functions. In Advances in experimental social psychology, vol. 43, pp. 115-155. New York, NY: Academic Press. (10.1016/S0065-2601(10)43003-8)

[40] Monin B, Miller DT. 2001 Moral credentials and the expression of prejudice. J. Pers. Soc. Psychol. **81**, 33. (10.1037/0022-3514.81.1.33) 11474723

[41] Catlin JR, Wang Y. 2013 Recycling gone bad: when the option to recycle increases resource consumption. J. Consumer Psychol. **23**, 122-127. (10.1016/j.jcps.2012.04.001)

[42] Ma B, Li X, Jiang Z, Jiang J. 2019 Recycle more, waste more? When recycling efforts increase resource consumption. J. Cleaner Prod. **206**, 870-877. (10.1016/j.jclepro.2018.09.063)

[43] Truelove HB, Yeung KL, Carrico AR, Gillis AJ, Raimi KT. 2016 From plastic bottle recycling to policy support: an experimental test of pro-environmental spillover. J. Environ. Psychol. **46**, 55-66. (10.1016/j.jenvp.2016.03.004)

[44] Hills TT, Todd PM, Lazer D, Redish AD, Couzin ID, Cognitive Search Research Group. 2015 Exploration versus exploitation in space, mind, and society. Trends Cogn. Sci. **19**, 46-54. (10.1016/j.tics.2014.10.004) 25487706 PMC4410143

[45] Berger-Tal O, Nathan J, Meron E, Saltz D. 2014 The exploration-exploitation dilemma: a multidisciplinary framework. PLoS ONE **9**, e95693. (10.1371/journal.pone.0095693) 24756026 PMC3995763

[46] Toyokawa W, Kim HR, Kameda T. 2014 Human collective intelligence under dual exploration-exploitation dilemmas. PLoS ONE **9**, e95789. (10.1371/journal.pone.0095789) 24755892 PMC3995913

[47] Katz K, Naug D. 2015 Energetic state regulates the exploration–exploitation trade-off in honeybees. Behav. Ecol. **26**, 1045-1050. (10.1093/beheco/arv045)

[48] Mao H, Alizadeh M, Menache I, Kandula S. 2016 Resource management with deep reinforcement learning. In Proc. of the 15th ACM workshop on hot topics in networks, pp. 50-56. New York, NY, USA: Association for Computing Machinery. (10.1145/3005745.3005750)

[49] Mason K, Grijalva S. 2019 A review of reinforcement learning for autonomous building energy management. Comput. Elect. Eng. **78**, 300-312. (10.1016/j.compeleceng.2019.07.019)

[50] Yogeswaran M, Ponnambalam SG. 2012 Reinforcement learning: exploration–exploitation dilemma in multi-agent foraging task. Opsearch **49**, 223-236. (10.1007/s12597-012-0077-2)

[51] Frankenhuis WE, Panchanathan K, Barto AG. 2019 Enriching behavioral ecology with reinforcement learning methods. Behav. Processes **161**, 94-100. (10.1016/j.beproc.2018.01.008) 29412143

[52] Dalamagkidis K, Kolokotsa D, Kalaitzakis K, Stavrakakis GS. 2007 Reinforcement learning for energy conservation and comfort in buildings. Build. Environ. **42**, 2686-2698. (10.1016/j.buildenv.2006.07.010)

[53] Creutzig F et al. 2022 Demand-side solutions to climate change mitigation consistent with high levels of well-being. Nat. Clim. Change **12**, 36-46. (10.1038/s41558-021-01219-y)

[54] Mundaca L, Ürge-Vorsatz D, Wilson C. 2019 Demand-side approaches for limiting global warming to 1.5°C. Energy Effic. **12**, 343-362. (10.1007/s12053-018-9722-9)

[55] Bajželj B, Richards KS, Allwood JM, Smith P, Dennis JS, Curmi E, Gilligan CA. 2014 Importance of food-demand management for climate mitigation. Nat. Clim. Change **4**, 924-929. (10.1038/nclimate2353)

[56] Lenzen M, Key*β*er L, Hickel J. 2022 Degrowth scenarios for emissions neutrality. Nature Food **3**, 308-309. (10.1038/s43016-022-00516-9) 37117569

[57] Buhl J, Acosta J. 2016 Indirect effects from resource sufficiency behaviour in Germany. In Rethinking climate and energy policies: new perspectives on the rebound phenomenon, pp. 37-54. Cham, Switzerland: Springer.

[58] Noblet CL, McCoy SK. 2018 Does one good turn deserve another? Evidence of domain-specific licensing in energy behavior. Environ. Behav. **50**, 839-863. (10.1177/0013916517718022)

[59] Seebauer S. 2018 The psychology of rebound effects: explaining energy efficiency rebound behaviours with electric vehicles and building insulation in Austria. Energy Res. Soc. Sci. **46**, 311-320. (10.1016/j.erss.2018.08.006)

[60] Tiefenbeck V, Staake T, Roth K, Sachs O. 2013 For better or for worse? Empirical evidence of moral licensing in a behavioral energy conservation campaign. Energy Policy **57**, 160-171. (10.1016/j.enpol.2013.01.021)

[61] Höök M, Bardi U, Feng L, Pang X. 2010 Development of oil formation theories and their importance for peak oil. Mar. Pet. Geol. **27**, 1995-2004. (10.1016/j.marpetgeo.2010.06.005)

[62] Segovia-Martin J, Creutzig F, Winters J. 2023 School-of-Collective-Intelligence/Jevons-Paradox-and-Cultural-Evolution. *Github Repository. See* https://github.com/School-of-Collective-Intelligence/Jevons-Paradox-and-Cultural-Evolution.

[63] Segovia-Martin J, Creutzig F, Winters J. 2023 A simulation tool can be accessed via the following links: https://jevons-collectiveintelligence.pythonanywhere.com/ or https://jsegoviamartin.pythonanywhere.com/.

[64] Segovia-Martin J, Creutzig F, Winters J. 2023 Data from: Efficiency traps beyond the climate crisis: exploration–exploitation trade-offs and rebound effects. Dryad Digital Repository. (10.5061/dryad.qjq2bvqnk). PMC10505854 37718604

[65] Segovia-Martin J, Creutzig F, Winters J. 2023 Data from: Efficiency traps beyond the climate crisis: exploration–exploitation trade-offs and rebound effects. Dryad Digital Repository. (https://doi.org./10.5061/dryad.bnzs7h4h4). 10.1098/rstb.2022.0405 PMC10505854 37718604

